# Deciphering the influence of NaCl on social behaviour of *Bacillus subtilis*

**DOI:** 10.1098/rsos.240822

**Published:** 2024-09-18

**Authors:** Prem Anand Murugan, Muktesh Kumar Sahu, Manish Kumar Gupta, T. Sabari Sankar, Sivasurender Chandran, Saravanan Matheshwaran

**Affiliations:** ^1^ Department of Biological Sciences and Bioengineering, Indian Institute of Technology, Kanpur, India; ^2^ Soft and Biological Matter Laboratory, Department of Physics, Indian Institute of Technology, Kanpur, India; ^3^ School of Biology, Indian Institute of Science Education and Research, Thiruvananthapuram, India; ^4^ Centre for Environmental Sciences and Engineering, Indian Institute of Technology, Kanpur, India; ^5^ Mehta Family Centre for Engineering in Medicine, Indian Institute of Technology, Kanpur, India

**Keywords:** biofilm, surface motility, surfactin, flagella, *Bacillus subtilis*, NaCl

## Abstract

Various environmental signals, such as temperature, pH, nutrient levels, salt content and the presence of other microorganisms, can influence biofilm’s development and dynamics. However, the innate mechanisms that govern at the molecular and cellular levels remain elusive. Here, we report the impact of physiologically relevant concentrations of NaCl on biofilm formation and the associated differences in an undomesticated natural isolate of *Bacillus subtilis*. NaCl exposure and its uptake by bacterial cells induced substantial changes in the architecture of pellicle biofilm and an upsurge in the expansion of biofilm colonies on agar surfaces. We have observed the upregulation of genes involved in motility and the downregulation of genes involved in the biosynthesis of extracellular matrix components through the transcription factor *sigD,* suggesting the possible underlying mechanisms. To further support these observations, we have used Δ*sigD and* Δ*srfAC* null mutants, which showed compromised NaCl-induced effects. Our results indicate that NaCl induces a lifestyle shift in *B. subtilis* from a sessile biofilm state to an independent unicellular motile state. Overall, we present evidence that NaCl can reprogramme gene expression and alter cellular morphology and the state of cells to adapt to motility, which facilitates the expansion of bacterial colonies.

## 1. Introduction

Bacteria are the most abundant and diverse forms of life on the planet [1]. They often exist as complex, structurally organized multicellular-like communities called biofilms. Biofilms thrive within self-produced, viscoelastic fluids rich in a variety of macromolecules such as exopolysaccharides (EPS), extracellular DNA and a range of proteins. These components are crucial in stabilizing the biofilms [2–7]. The sessile multicellular state of the biofilm provides various fitness advantages to the microbes by increasing their tolerance to external stressors [2–7]. Due to this enhanced tolerance, biofilms are major threats in various sectors like healthcare (causing chronic infections and anti-drug resistance) [8–11] and in industrial sectors, including agriculture, bioremediation, biofouling and food hygiene [12–14]. Moreover, biofilms are essential in aiding food digestion, biogeochemical processes, plant growth and biodegradation [15–20]. Thus, given the importance and impact of biofilms, it is imperative to gain a comprehensive understanding of them to limit their threat and expand on their promises.

Bacterial biofilms are a result of the interplay between chemical, biological and physics principles that help establish intra- and inter-species communication across colonies [21–26]. The development of biofilms relies on intricate interactions among biochemical, cellular (involving changes in gene expression), and mechanical processes (including cell-to-cell interactions, nutrient diffusion and hydrodynamics interactions) [24,27–34]. As bacterial cells proliferate inside a self-secreted viscoelastic environment of EPS, they form the biofilm, creating three-dimensional architectures due to in-plane compressive stress [35–38].

Despite the vast knowledge, there is still a dearth of understanding on several critical aspects of the biofilm lifestyle essential for bacterial survival and transmission. Importantly, the switching between the sessile (biofilm) to motile states is regulated by complex and diverse mechanisms depending on the environmental signals, effectors, and signal transduction, which are yet to be well understood [39]. Additionally, the impact of chemical-mediated changes in motility mechanisms [40,41] that presumably alter the timely expression of crucial genes that are responsible for biofilm formation and motility is poorly understood. As understanding motility can be one of the key factors in unravelling the response of bacteria exposed to chemicals, it is imperative to identify the correlations between the chemical cues and the genetic, physiological and molecular pathways that can activate and mediate motility.

*Bacillus subtilis* requires the production of a lipopeptide, named surfactin, to induce biofilms and move over solid surfaces [42–44]. Surfactin facilitates the *B. subtilis* cells to colonize new environments by sliding (flagella independent) or swarming motility (flagella dependent) by increasing the surface wettability of the solid surfaces [42–44]. Earlier studies on *B. subtilis* in response to NaCl have only focused on the high and stress-inducing concentrations of NaCl [45–48] and showed that there is a decrease in motility of *B. subtilis* at such high concentrations [45–48]. In *B. subtilis,* under high salt conditions, most of the chemotaxis and motility-related genes are repressed. Out of the repressed genes under salt stress, most genes happen to be under the *sigD* regulon [47,48]. The *sigD* regulon controls the major class of genes under motility and chemotaxis, including genes under *sigB* regulon (central regulator of the general stress response), thereby indirectly regulating the sessile biofilm state [47,49,50].

However, in natural environments like soil and association with plants, the maximum salinity (NaCl) experienced by *B. subtilis* is generally less than 2% by weight [45,51–54]. Hence, to understand the effect of NaCl at these physiological and naturally relevant concentrations (≤ 2%) on biofilm and motility, we generated knockout mutants of the two genes *sigD* and *srfAC*. The knockout mutants of *sigD* (stress-responsive sigma factor) and *srfAC* (part of surfactin operon) revealed the role of NaCl in biofilm formation and dynamics. To summarize, we elucidate the role of NaCl as a key player in shaping biofilm architecture, cellular morphology and gene expression patterns, triggering significant shifts in behaviour and functionality. The study illustrates how a simple chemical signal can initiate and regulate the shift of cells from a stationary biofilm state to individual motile cells.

## 2. Experimental procedures

### 2.1. Bacterial strains and media

The natural isolate *B. subtilis* IITKSM1 strain was used in this study. The strain isolation and sequencing information are shown in our earlier work [46]. The *B. subtilis* IITKSM1 was grown on a rich medium (2% peptone, 1% yeast extract and 2% dextrose). In addition to the rich medium, different NaCl concentrations ranging from 0 to 2 wt.%, amounting to a maximum molarity of *ca*. 0.37 M, were used. The pellicle formation and colony architecture assays were performed in rich media and varied concentrations of NaCl, as described above. Complementary experiments were performed using minimal media glutamate glycerol (MSgg) broth as used in [55] or on MSgg media supplemented with 1.5% agar and with suitable concentrations of NaCl. All medium components were made as solutions in sterile MQ water and sterilized by either autoclaving or filter sterilization and mixed aseptically before use. The culture was maintained on rich and LB Agar plates. The primers used for the study are given in table 1.

**Table 1.** Primers used for performing Q-PCR in *B. subtilis* IITKSM1 in the presence and absence of NaCl.

TapAFW	5'AACCGACAGTCCCTAAAAAAG3'
TapARE	5'CCTTCTGGTCTGATTGAGTTTC3'
Spo0AFW	5'TTCAACACAACCGCAAGCC3
Spo0ARE	5'TTTCCTCTGCTCCATGCCAC3'
RpsEFW	5'ACTGGAGTTATCGCTGGAGG3'
RpsERE	5'AGTGTTGCACGAATCATGTTG3'
RpsJFP	5'CGTGAGCAATTTGAAATGCG3'
RpsJRP	5' GATATCGACACCAGATGGTAAG3'
SwrAFW	5'TCAGCTACAAAAGCACTAAGTC3'
SwrARE	5'ATGTTTGGCGATTCCTC3'
CheWFW	5'CTTCATCCACAATCCAGCC3'
CheWRE	5'GGGGTAATCAATTTACGCGG3'
EpsEFW	5'TATGATGAGAGCGAGTGCC3'
EpsERE	5'GCTTCCTGAAGATTATAGCC3'
FlgMFW	5'AAAAACTGCTGCACAGCC3'
FlgMRE	5'GCGTCTACTTTGTATGACCC3'
HagFW	5'TCAAACCAACGTGCTAAACTTG3'
HagRE	5'AAATCGCTCATTTCTTTCGCC3'
SinIFW	5'TTAATGGTGGAAGCCAAAGAG3'
SinIRE	5'GGATTTACGGTATGACTTCTG3'

### 2.2. Generation of knockout mutants

To generate the mutants of *B. subtilis* IITKSM1, the mutant library of *B. subtilis* 168 was utilized. The genomic DNA of the mutants of *B. subtilis* 168 (*sigD* (BKK16470), *srfAC* (BKK03510) and *epsE* (BKK34330)) was isolated, and PCR amplification of approximately 1.5 kb upstream and downstream sequences of the *kan^r^* cassette was performed. The amplified PCR fragment was transformed into *B. subtilis* strain*,* IITKSM1 was selected for mutant strains on the LB-Kanamycin plate, which was confirmed by PCR and sequencing [56]. The primers used for generation of knockout mutants are listed in table 2.

**Table 2.** Primers used for performing knockout mutants in *B. subtilis* IITKSM1.

MKS_33	GCTTCGAAATCGTGGATCTG	*srfAC* knockout
MKS_34	TTCATCCCAAAGACGAGG	
MKS_35	ATTCATCAGCCAAAGGAGTG	*epsE* knock out
MKS_36	CCATGCTGAACAAATCTTTCC	
TS402	GGCAGTCCCTCTTATCACC	*sigD* knockout
TS403	GGAATCAAAGAAGAGCAGATGGAC	

### 2.3. Pellicle formation assay

**Figure 1.**
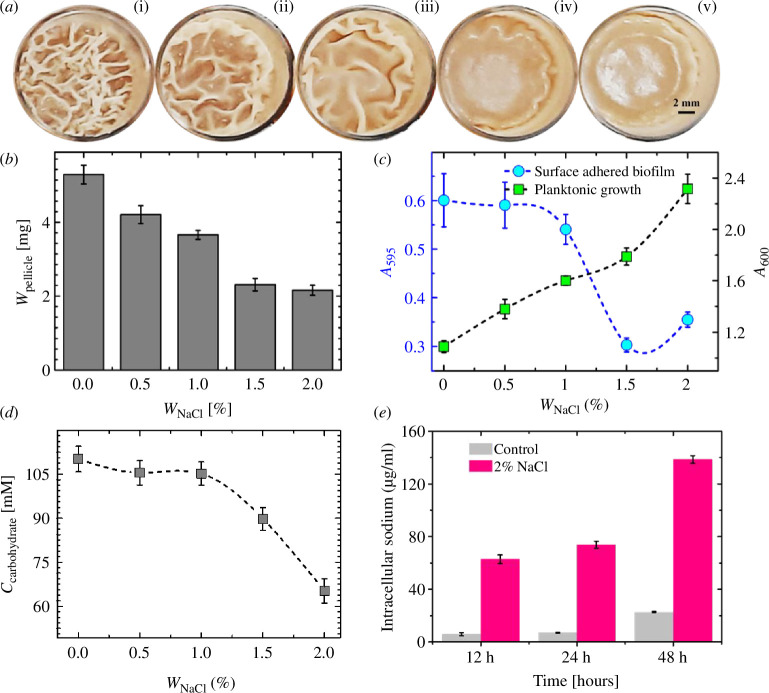
Pellicle formation of *Bacillus subtilis* and quantification in static liquid media. (*a*) Top view of pellicles of *B. subtilis* IITKSM1 (wild-type) under different NaCl concentrations [(*i*)*W*_NaCl_ = 0% (*ii*) *W*_NaCl_ = 0.5% (*iii*) *W*_NaCl_ = 1% (*iv*) *W*_NaCl_ = 1.5% (*v*) *W*_NaCl_ = 2%] in rich liquid culture media at *ca*. 48 h of incubation at 30°C (24 well plate, well diameter—15.5mm). All images are contrast-enhanced for better visibility. (*b*). Dry weight of pellicles (*W*_pellicle_) as a function of *W*_NaCl_ (*n* = 9, triplicates with three independent experiments). The data plotted is the mean obtained from three independent experiments. (*c*) Optical density was measured for wild-type planktonic cells (*A*_600_) and crystal violet-stained surface-adhered biofilm (*A*_595_) as a function of *W*_NaCl_ (*n* = 9) after 48 h. (*d*) Secreted carbohydrate concentration was estimated using the phenol-sulphuric acid method for varying NaCl concentrations (*n* = 9, triplicates with three independent experiments) after 48 h. (*e*). Intracellular sodium concentration in the absence and presence of 2 wt.% NaCl as measured by ICP-MS (triplicates). Standard error (s.e.) = [stdev/sqrt (count)]. *W*_NaCl_ represents the amount of NaCl in the media by weight percentage.

For floating pellicle assays, *B. subtilis* IITKSM1 was grown in either rich or minimal medium in Tarsons (24 well) plates at 30°C for 48 h and 72 h, respectively. The NaCl concentrations were varied in each well [28]. The number of pellicles, pellicle fold width and area of folds in the pellicle were analyzed using ImageJ software [57] and plotted using Origin Pro 9.1 version (Origin Lab Corporation [http://www.OriginLab.com]). To quantify the dry weight of the formed pellicle, 1 ml of 100% ethanol was carefully poured under the pellicle using a pipette to lift it from the surface of the liquid. The obtained pellicles were vacuum-dried, and the weights were measured. The data shown in figure 1 are representative of three independent experiments.

### 2.4. Surface motility assay

Bacteria were cultured in rich medium at 30°C for 12 hours at 200 rpm. The inoculum was adjusted to obtain OD 600 nm approximately 1.0. Three microliters of cell suspension were spotted on 1.2% agar-rich medium without and with 2 wt.% NaCl and grown at 30°C for up to 48 h. The time-lapse photos were captured using a mobile camera with a Sony image sensor (16MP (f1.7)). A similar procedure was followed to observe the colony morphology in minimal MSgg media with varying NaCl concentrations.

### 2.5. Disc engulfment assay

The extent of engulfment by *B. subtilis* IITKSM1 with and without NaCl was performed using methods discussed in reference [58]. Three PVDF membrane discs of 6 mm diameter were placed at 1.5, 3 and 4.5 cm from the inoculated biofilm colony. For each *B. subtilis* IITKSM1 colony, the number of PVDF discs engulfed by the expanding biofilm colony was captured and quantified. The results presented here are the measurements of at least four different colonies at each time point.

### 2.6. Optical microscopy and imaging of motility

*B. subtilis* IITKSM1 motility on the surface of rich agar medium (1.2%) was imaged with a time-lapse optical microscope (Olympus BX40 microscope) using 100× magnification (Olympus, Japan). Optical micrographs of the expanding edges, in the presence and absence of NaCl, were obtained *ca*. 14 h after spotting on the agar plate.

### 2.7. Image analysis and bacteria tracking

Image analysis and cell-tracking were performed using the open-access package CellProfiler (CellProfiler Project [http://www.cellprofiler.org]). The input images were first converted to grayscale images, whose contrast is enhanced by implementing an inbuilt module named ‘EnhanceOrSuppressFeatures’ in the CellProfiler. Using the contrast-enhanced image, we obtained the binary image by implementing the ‘otsu-thresholding method’. Thresholded images, as obtained, are classified into cells and backgrounds using the watershed algorithm. Cell orientation and its dimension were calculated by using the ‘objectSizeShape’ module in CellProfiler. Such images were subsequently used to track the trajectory of individual cells using a standard particle-tracking algorithm based on a ‘Follow neighbour criterion’ in successive frames. Further, the trajectories were analyzed with Python scripts to obtain mean square displacements.

### 2.8. Surface profilometer

Optical profilometry was performed and acquired using a non-contact optical profilometer (Bruker GT-KO, USA). Images were captured from the centre of bacterial colonies on agar with an objective of 50× magnification. The resultant surface images with an area of 240 × 180  µm up to 0.3–0.5 µm were evaluated with the Vision64 software.

### 2.9. Analysis of surface wettability

The *B. subtilis* IITKSM1 cells were grown for 48 h on a rich agar medium (1.2% agar). The contact angle measurement was determined using a goniometer (KRUSS-Drop Shape Analyzer-DSA 25E). The water droplet was placed close to the proximity of the mature biofilm centre, and the contact angles were measured for different samples. The drop profile was processed using the image analysis package ADVANCE software, KRUSS, GmbH, Germany.

### 2.10. Transmission electron microscopy (TEM)

For investigation of flagella by transmission electron microscopy (TEM) [59], *B. subtilis* IITKSM1 was grown for 12 h in a rich medium without and with 2% NaCl supplementation. The cells grown on rich medium broth for *ca*. 14 h were absorbed onto copper grids. The grids were washed twice with PBS. Negative staining of the cells was done using 1% freshly prepared uranyl acetate. Samples were viewed in FEI Technai G2 20 twin TEM.

### 2.11. RNA isolation and Q-PCR

RNA was isolated from the 12 h shaking culture of *B. subtilis* IITKSM1 grown at 37°C. The procedure was followed from [60]. Briefly, 10 ml of 12 h culture was centrifuged and pelleted. The pellet was frozen and stored at − 80°C for 24 h. The pellet was later dissolved in 0.5 ml of lysis buffer (30 mM Tris, 10 mg/ml lysozyme and 10 mM EDTA) and kept at 37°C for 30 min. 1 ml of Trizol reagent and 0.3 ml of chloroform was added to the tube and centrifuged at 15000 rpm for 20 min at 4°C. The aliquoted top aqueous layer was treated with isopropanol and stored in the refrigerator (−20°C) for 2 to 4 h. The precipitated RNA was centrifuged at 15 000 rpm for 20 min at 4°C. The obtained pellet was washed with 0.5 ml of ice-cold 70% ethanol and air dried and finally, the pellets were suspended in 20 µl Milli-Q water. The isolated RNA (2 µg) was converted to cDNA with a Quantitect Reverse Transcription kit (Cat. No. 205311). The genes chosen were *epsE*, *tapA, spo0A, swrA, srfAA, flgM* and *cheW,* with *rpsE and rpsJ* as housekeeping genes. Real-time PCR was performed using Promega GoTaq Green Q-PCR Master mix (A6001), one step with the following conditions for activation of the enzyme at 95°C for 30 s, 40 cycles of denaturation at 95°C for 30 s, annealing extension at 55°C for 1 min. The melt curve analysis was performed at 65°C for 1 min in CFX connects TM Real-time PCR detection system (Bio-Rad, USA). The primers used are listed in table 1. Relative mRNA levels were determined by fold change as described [61].

### 2.12. Quantification of planktonic cells, surface adhered biofilm formation and amount of secreted carbohydrates

To determine how the planktonic cells and surface-adhered biofilms alter under different NaCl concentrations, the *B. subtilis* IITKSM1 was grown in Tarsons (96 well) plates. The cells were grown for up to 48 h to form biofilms in the standardized rich medium at 30°C as static culture. The surface-adhered biofilms were subjected to staining after removing the pellicles and spent media and replacing the wells with 200 µl of 1% crystal violet for 30 min at room temperature followed by washing with distilled water. The stained crystal violet was resuspended in either 95% ethanol or 70% ethanol and 30% acetic acid. The absorbance was measured at 595 nm [62]. The secreted carbohydrate concentrations were measured from the culture filtrate. The concentrations were determined as previously [63]. The data represented here are from three independent experiments.

### 2.13. Estimation of intracellular concentration of sodium in *B. subtilis* IITKSM1 cells

Bacterial cells with balanced (equal weight) growth were obtained from 0 to 2% NaCl-supplemented (10 ml) rich media (yeast extract, peptone and dextrose) at different time intervals. The cells were pelleted from 5 ml after 12, 24 and 48 h time points, and the excess media was removed. The obtained pellets were washed using 1M Tris-Cl to remove excess ions and media without cell lysis. The centrifugation was done at 13 000 rpm for 2 min. The cell samples were instantly freeze-dried. The samples were lysed in 2 ml 1% HNO_3_, followed by sonication to break up the lysate. The sonication was done in a bath sonicator at room temperature for a minimum of 5 min. The total sodium ions were estimated in diluted samples analyzed by ICP-MS (Thermo iCAP-Qc). The Dilution factor used was 200 for the analysis. The external standard was applied for quantification by Qtegra™ Intelligent Scientific Data Solution™ (ISDS) software [64].

### 2.14. Fourier transform infrared spectroscopy (FTIR)

A mature pellicle of *B. subtilis* IITKSM1, grown for *ca*. two days on the rich medium (with and without NaCl) was cautiously separated. The pellicle was dried and lyophilized. Infrared spectroscopic measurements of the samples were performed on a Bruker Tensor 27 IR spectrophotometer (Bruker Corporation, USA, KBr Beam splitter). All spectral readings were smoothed using the standard automatic smooth function [65].

## 3. Results and discussion

### 3.1. NaCl-mediated regulation of pellicle and surface-adhered biofilm formation

The pellicle formation assay is a suitable method to study the effect of various environmental chemical cues on *B. subtilis* biofilm formation in liquid cultures. Figure 1 summarizes NaCl-induced changes in the formation of pellicles. A systematic increase in the concentration of NaCl (represented as *W*_NaCl_) from 0 to 2 wt.% showed drastic changes in the architecture of pellicles of wild type (figure 1*a*; electronic supplementary material, figure SF1 and 2). To understand the observed change, we measured the number of wrinkles (*N*wrinkles) and the area (*A*) covered by them (electronic supplementary material, figure SF3A). The number of wrinkles and the area covered by the wrinkles are decreased with an increase in NaCl concentration (*W*_NaCl_). Studies have shown that the wrinkled appearance of pellicles arises from the *in-plane* compressive stress caused by the confined geometry (such as the wells of the culture plates) where the cells are growing [66]. The *in-plane* stress here refers to the load forces exerted (parallel) to the pellicle biofilm by the walls of the wells where the *B. subtilis* cells are grown. There was a gradual decrease in pellicle formation with an increase in concentrations of NaCl in the media. As presumed, there is an overall decrease in the dry weight of the pellicle with an increase in concentration of NaCl (figure 1*b* and electronic supplementary material, SF3B). The growth rate of bacteria (electronic supplementary material, figure SF4) and the chemical nature of certain biomolecules in the pellicle remained the same, as shown by FT-IR spectroscopy (electronic supplementary material, figure SF5). Other than the pellicle biofilm, another type of biofilm that is predominantly formed by *B. subtilis* is the surface-adhered biofilm. Therefore, next, we checked the consequence of NaCl exposure on the surface-adhered biofilm and compared it with pellicle biofilms. NaCl induced the reduction of the surface-adhered biofilm and the associated increase in planktonic growth of bacteria, as measured via the optical densities at 595 nm (crystal violet assay) and 600 nm (growth density), respectively (figure 1*c*). These findings, taken together, indicated that there is a decrease in the *in-plane* stresses (load force exerted by the well walls parallel to the biofilm as it grows) developed in the pellicles in the wild type. These forces continue to increase the *in-plane* stress on the pellicle biofilm as the pellicle continues to expand. We imply that this *in-plane* stress results in the formation of wrinkles on the pellicle biofilm. To explore why the *in-plane* stress decreases with an increase in concentrations of NaCl, we measured the concentration of secreted carbohydrates (represented as *C*_carbohydrate_), the most important component of EPS that contribute to viscoelastic properties in pellicle biofilms [66]. We observed a simultaneous decrease in the dry weight of the pellicle (*W*_pellicle_), surface-adhered biofilms and a drop in the concentration of secreted carbohydrates (*C*_carbohydrate_) with an increase in the concentration of NaCl (*W*_NaCl_) as shown in figure 1*d*. These findings shed light on the potential causes behind NaCl-induced changes in biofilm architecture. The overall reduction of the dry weight of the pellicle (*W*_pellicle_) led to reduced *in-plane* stress of the pellicle. Further, the decrease in the concentration of the secreted carbohydrates suggests a possible decrease in the overall viscoelasticity of the pellicles with an increase in the concentration of NaCl. The decrease in the number of wrinkles in the pellicles with an increase in the concentration of NaCl in the media (electronic supplementary material, figure SF3A) can be attributed to buckling instability. The decrease in modulus of the pellicle (monolayer) is expected to result in a decrease of the wavelength and, in turn, in a decreased number of wrinkles. These observations collectively demonstrate the NaCl impact on the three-dimensional architecture of pellicles. To determine whether the observed phenotypic changes were due to the increase in the intracellular concentrations of NaCl, we used ICP-MS to measure the concentration of sodium ions inside the cells. We have found that sodium ions are significantly increased after 12, 24, and 48 h when exposed to 2 wt.% NaCl (figure 1*e*), indicating approximately a sixfold greater sodium uptake. This suggests that the heightened intracellular sodium levels due to NaCl treatment could be a key factor influencing *B. subtilis* biofilm dynamics.

### 3.2. Regulation of biofilm and motile transition in the presence of NaCl

**Figure 2.**
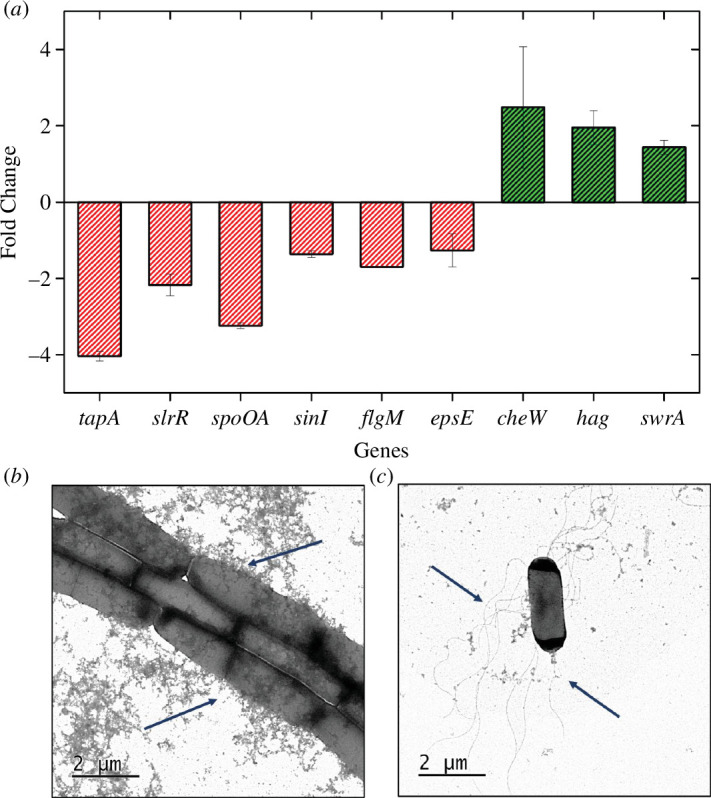
Mutually exclusive lifestyle between biofilm and planktonic state of *B. subtilis* IITKSM1 under the influence of NaCl. (*a*) Changes in the gene expression level of motility and biofilm-inducing genes in the presence of NaCl (2 wt.%) compared to cells grown without NaCl (*n* = 9, triplicates with three independent experiments). (*b*) TEM images showing cells grown without NaCl. (*c*) Cells grown with 2 wt.% NaCl (right) in liquid media. The dark blue arrows (*b*) indicate cell chaining, and the dark blue arrows (*c*) point towards the flagella. Standard error (s.e.) = [stdev/sqrt (count)].

We were curious about how the uptake of Na^+^ by cells translates into molecular-level changes. To investigate this, we measured the relative change in gene expression upon adding NaCl. Using qRT-PCR, we examined the levels of specific motility and biofilm-related genes. The cells were grown for approximately 12 h to reach high density and approach the stationary phase. At this phase, the cells regulate their gene expression to form biofilms [67]. As seen in (figure 2*a*), the expression of *tapA* (biofilm assembly accessory factor) is decreased up to fourfold. Similarly, there is a threefold and twofold decrease in the master regulator of biofilm *spo0A* and *slrR,* respectively. There is a twofold reduction in *epsE* (molecular clutch), the matrix-producing gene. The reduction in the expression of *slrR spo0A*, *tapA* and *epsE*, in turn, impacts (diminishes) the formation of pellicles and biofilms. There is a threefold reduction in *flgM*, a negative regulator of flagellar biosynthesis. Moreover, there is a twofold increase in the swarming gene *swrA* and chemotaxis gene *cheW* in the presence of NaCl, which likely contributes to the increased cellular motility. Further, a twofold increase in the expression of the *hag* (flagellin) gene. The NaCl treatment increased the expression of motility-associated genes and decreased the expression of biofilm-related genes, notably *slrR* and *epsE* [42,43]. This highlights NaCl’s role in regulating the transition from sessile to motile states. To further visualize the effects on individual cells, we performed transmission electron microscope (TEM) measurements of cells grown on a liquid medium with and without NaCl. As seen in figure 2*b*, in the absence of NaCl, most of the cells were chained, while in the presence of NaCl (figure 2*c*), the cells were separated and showed decreased chaining. Strikingly, we observed that in the presence of NaCl, most of the cells (approx. 52% cells were flagellated compared to approx. 10% cells in control *n* = 150) were abundantly flagellated, in contrast to the non-flagellated cells in the absence of NaCl.

### 3.3. Role of *sigD* regulon in NaCl-Induced changes in biofilm formation and flagellation in *B. subtilis*

**Figure 3.**
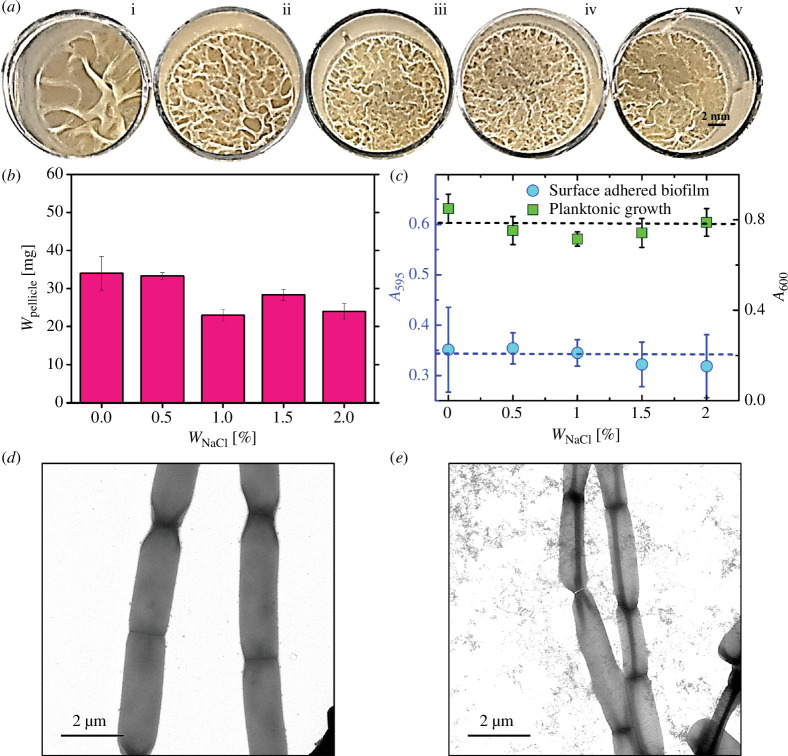
Pellicle formation of Δ*sigD* strain of *B. subtills* and quantification in static liquid media. (*a*) Top view of pellicles of Δ*sigD B. subtilis* IITKSM1 under different NaCl concentrations [(*i*)*W*_NaCl_ = 0% (*ii*) *W*_NaCl_ = 0.5% (*iii*) *W*_NaCl_ = 1% (*iv*) *W*_NaCl_ = 1.5% (*v*) *W*_NaCl_ = 2%] in rich liquid culture media after *ca*. 48 h of incubation at 30°C (24 well plates, well diameter—15.5 mm). All images are contrast-enhanced for better visibility. (*b*) Dry weight of pellicles (*W*_pellicle_) as a function of *W*_NaCl_ (*n* = 9) after 48 h. The data plotted is the mean obtained from triplicates with three independent experiments. (*c*) Optical density was measured for wild-type planktonic cells (*A*_600_) and crystal violet-stained surface adhered biofilm (*A*_595_) as a function of *W*_NaCl_ (*n* = 9) after 48 h. (*d*) TEM images showing Δ*sigD* cells grown without NaCl. (*e*) Δ*sigD* cells grown with 2 wt.% NaCl (right) in liquid media. Standard error (s.e.) = [stdev/sqrt (count)]. *W*_NaCl_ represents the amount of NaCl in the media by weight percentage.

One of the strong regulators of stress response genes, especially associated with salinity stress in *B. subtilis*, is *sigD* regulon [47]. It has been shown that high salinity (1.5M NaCl) stress induces genes involved in motility. Therefore, we wanted to test the role of *sigD* in our observed phenotypes under our conditions (0–2 wt% NaCl). We have found that in the case of Δ*sigD* mutant, no significant systemic changes in pellicle architecture were observable from 0 to 2 wt.% (figure 3*a*; electronic supplementary material, figure SF6). We quantified the deviations of overall biomass (dry weight) of pellicles (figure 3*b*) and surface-adhered biofilms Δ*sigD* mutant (figure 3*c*) on rich media from 0 to 2 wt.%. We found no significant change with an increase in NaCl concentration, as observed with the WT cells. This confirmed that the loss of *sigD* negated the effect of NaCl on pellicle formation. As *sigD* regulon is a known regulator of flagellar synthesis, we investigated the effect of *sigD* deletion on flagellation with and without NaCl. As seen in figure 3*d,e*, no flagella were observed in any of the cells, either in the presence or absence of NaCl. Based on these observations, we propose that the reduction in biofilm upon exposure to NaCl might be due to the reduction in chaining and increased flagellar synthesis through *sigD* regulation. In wild-type cells, this could be the result of a higher subpopulation of cells in a free unicellular motile state rather than in a sessile biofilm state compared to cells without NaCl exposure.

### 3.4. NaCl-mediated regulation of biofilm colony expansion on agar surface

**Figure 4.**
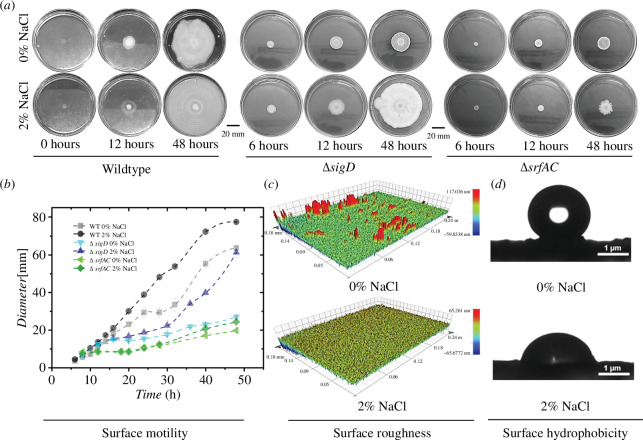
Biofilm colony expansion of *B. subtilis* on agar surface. (*a*) Representative time-lapse images showing the lateral expansion of wild type, **Δ** *sigD* and **Δ** *srfAC* cells in the absence and the presence of 2 wt.% NaCl (Plate diameter: 86 mm) (*b*) Diameter [mm] of the expanding biofilms (*n* = 9, triplicates with three independent experiments) on 1.2% rich agar media. (*c*) Profilometric images of wild-type biofilm colony centre grown without NaCl and with 2 wt.% NaCl, which clearly shows differences in height features *n* = 3. (*d*) Surface wettability of the colony centre grown without NaCl and with 2 wt.% NaCl (1.2% agar) is captured via the contact angles of water on the respective surfaces. Contact angles for 0% NaCl: 139.6° and 2% NaCl: 76.5°. Standard error (s.e.) = [stdev/sqrt (count)].

To further analyse the effects of NaCl on biofilm properties, we performed experiments on biofilms that were grown on an agar surface, and the results are shown in figure 4*a*. Noticeably, we observed an increase in the rate of lateral expansion of *B. subtilis* biofilms in the presence of 2 wt.% NaCl (figure 4*b*). We found that only limited concentrations of NaCl (*W*_NaCl_ < 2.5 wt.%) showed the increased surface motility of cells, while at higher concentrations, there is a decrease in surface motility (electronic supplementary material, figure SF7). This corroborated the earlier reports that showed a reduction in surface motility in the presence of NaCl [47,68–70].

To investigate the enhanced surface motility in the presence of NaCl further, we evaluated the ability of biofilms to engulf foreign objects [58]. For this purpose, we used PVDF membrane discs placed 1.5 cm apart along the lateral expansion direction. As expected, the biofilms in the presence of NaCl exhibited higher engulfing ability than the biofilms grown in the absence of NaCl (electronic supplementary material, figure SF8). The chosen low concentrations of NaCl allowed us to examine the increased motility of cells on the surface, revealing a novel characteristic of the cells in biofilms.

In pellicle biofilms, we observed a significant reduction in the wrinkles upon adding NaCl. To verify if such variations exist in the topography of biofilms grown on the agar surface, we performed surface profilometry and the results are summarized in figure 4*c*. Surface profilometry is a technique of choice as the mature biofilms are quite dense and can be visualized by an optical microscope. Assuming a chemical homogeneity at the lateral length scales of our profilometer analysis, the differences in the light absorption could be related to the mean roughness of the surface. Biofilm shows peripheral surface features of approximately 15  µm height without NaCl but no peripheral surface features in the presence of 2 wt.% NaCl.

The addition of NaCl showed a decrease in the surface coarseness of the biofilm, suggesting a reduction in the higher-order structure formation. This reduction in higher-order structures affects various properties of biofilms, such as hydrophobicity [71], susceptibility to penetration of foreign molecules [72] and tolerance to mechanical stressors [35,36,38]. Here, the wettability of the biofilm surface in the presence and absence of NaCl (figure 4*d*) has been captured. The contact angle of water at the centre of the biofilms in the presence of NaCl was found to be *approximately* 77°, in contrast to 140° for biofilms formed without NaCl. Thus, biofilms grown in the presence of NaCl showed a reduced hydrophobicity, which may, in turn, be harnessed to facilitate the penetration of foreign molecules like drugs into the biofilms.

#### 3.4.1. Surfactin’s role in NaCl-induced effects on biofilm and surface motility of *B. subtilis*

We observed that the *sigD* mutant growth in liquid media negated the NaCl-induced effects on biofilm. However, for surface motility/expansion (on agar), we did not see such an effect in the Δ*sigD* mutant strain. Upon deletion of the *srfAC* gene, we observed no increased surface motility with an increase in *W*_NaCl_ (figure 4*a*). These results revealed that *sigD* may play a more important role in response to NaCl in liquid media, while surfactin produced by the *srfAC* gene may have a greater impact on surface motility in the presence of increasing *W*_NaCl_ (figure 4*b*). These findings led us to question whether the presence of NaCl influences biofilm formation only at the macroscopic length scales or also at the cellular scales.

### 3.5. NaCl affects the kinetics of biofilm

**Figure 5.**
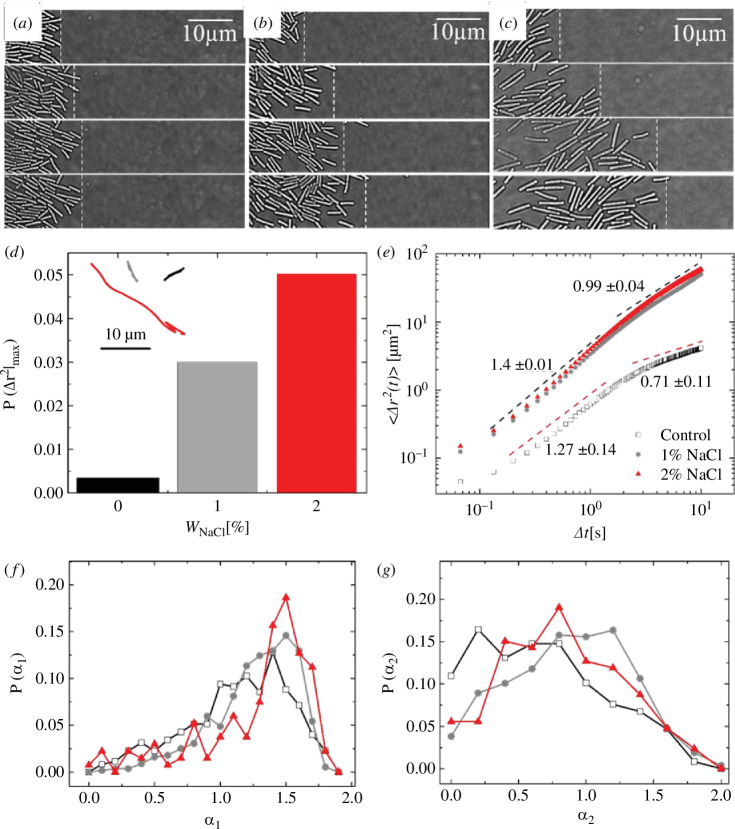
Optical micrographs and analysis of *B. subtilis* motility on agar with and without NaCl. Time series of optical micrographs every 2 min capturing the lateral expansion of biofilms at the cellular level in (*a*) the absence of NaCl, (*b*) in the presence of 1 wt.% NaCl and (*c*) 2 wt.% NaCl. Microscopy experiments were performed ca. 8 h after the inoculation of culture on the agar plates. (*d*) The probability that a cell has displaced at least 3.5 times larger than the length of the bacteria, i.e. P (Δr^2^|_max_) = ∑_i_ P_i_(Δr^2^|_max_), where √Δr^2^|_max_ > 3.5. Inset of (*d*) Representative trajectories (black and red correspond to the trajectories in the absence and presence of NaCl, respectively) of bacteria in both cases. (*e*) Mean square displacement of motile cells in the presence of 2 wt.% NaCl (red circles), 1 wt.% NaCl (grey circles), and in the absence (black squares) of NaCl. Distribution of exponents characterizing the dynamics of bacteria, in the presence and absence of NaCl, (*f*) for the initial regime (Δt < 3 s) and (*g*) the later regime (Δt > 3 s).

To investigate the influence of NaCl on *B. subtilis* at a cellular level, we examined the spatial and temporal expansion of colonies with and without NaCl using time-lapse optical microscopy. Our focus was on the growing edge containing a single layer of bacteria. In figure 5*a–c*, we present a series of optical micrographs showing biofilms with and without NaCl, taken approximately 8 h after inoculation on the agar surface. Consistent with the results from figure 4, we observed increased surface motility of bacteria in the presence of 1 and 2 wt.% NaCl. This observation is supported by the representative trajectories shown in the inset of figure 5*d*. To further quantify, we deduced mean squared displacements, <Δr^2^(t)>, from the trajectories of all bacterial cells and deduced the probability distribution of maximum displacements (electronic supplementary material, figure SF9). Interestingly, we observed that the fraction of cells showing zero displacements is higher in the absence of NaCl (electronic supplementary material, figure SF10) and the collective probability (P_large_) for displacements larger than at least 3.5 times the length of the bacteria increased systematically with an increase in W_NaCl_ (figure 5*d*). This supports an increased subpopulation of motile cells in the presence of NaCl, revealing a dynamic shift in the behaviour of bacteria. To understand the microscopic characteristics of cellular dynamics, in figure 5*e*, we show the temporal evolution of ensemble-averaged <Δr^2^(t) > in the presence of 1 and 2 wt.% NaCl, in comparison with the control sample containing no NaCl. Overall, there is an apparent increase in the motility of cells in the presence of NaCl, augmenting our earlier results. In figure 5*e*, we find two dynamic regimes differing in the exponent (<Δr^2^(t)> ∼ t^α^) characterizing the nature of microscopic dynamics. In the initial time regime, until ca. 3 s, the bacterial cells were displaced via super-diffusive motion in the presence and absence of NaCl, albeit with minor differences in the exponents: α_1_|_NaCl_ ≈ 1.4 and α_1_|_control_ ≈ 1.27.

Interestingly, after ca. 3 s, we observed a transition from super-diffusive behaviour to sub-diffusive behaviour (α_2_|_control_ ≈ 0.7) in the absence of NaCl, while the cells in the presence of NaCl displayed a transition from super-diffusive behaviour to diffusive behaviour (α_2_|_NaCl_ ≈ 1.0). While the super-diffusive behaviour of biological systems has been identified in various studies (3, 4), the observed transition from super-diffusive to diffusive (in the presence of NaCl) or sub-diffusive (in the absence of NaCl) motion is not directly apparent.

To understand these observations, we explored the values of the exponents α_1_ and α_2_, characterizing the dynamics of all the individual bacteria within the leading edge. The temporal evolution of <Δr^2^(t)> corresponding to all the motile cells is shown in electronic supplementary material, figure SF9, in comparison with the ensemble-averaged ones. Interestingly, as shown in figure 5*f* *,g*, the distribution in α_1_ and α_2_ became progressively narrower as we increased the concentration of NaCl. This suggests a systematic decrease in the extent of dynamic heterogeneity with the increase in NaCl. The smaller displacements and larger dynamic heterogeneity in the absence of NaCl indicate the existence of subpopulations of cells that are not motile, which may cause crowding of the cells. This, in turn, may result in the slowing down of dynamics, as we have observed via the transition from super-diffusive motion to sub-diffusive motion. In addition, as shown earlier, there is a larger secretion of EPS (carbohydrate) in the absence of NaCl. This may further impose limitations on the dynamics of bacteria in the absence of NaCl and support the observed transition from super-diffusive to sub-diffusive motion. Moreover, the reduction in the concentration of secreted EPS is expected to fluidize the biofilm in the presence of NaCl. This, in addition to the reduced dynamic heterogeneity, may result in the observed transition from super-diffusive to diffusive behaviour in the presence of NaCl. Moreover, it may be possible that the bacterial cells secrete other compounds in the presence of NaCl, which might underlie the enhanced dynamics of bacterial cells.

## 4. Conclusions

Here, we investigated the influence of common salt (NaCl, a strong inducer of stress response in bacterial behaviours at high concentrations [47,68,72,73]) on complex structure formation, dynamics of biofilm, flagella synthesis and colony expansion in an undomesticated strain of *B. subtilis* IITKSM1 [74].

We have characterized the intricate relationship between NaCl exposure and the behaviour of *Bacillus subtilis* biofilm at both macroscopic and cellular levels. *B. subtilis* is a gram-positive, motile, spore-forming, non-pathogenic bacteria that is extensively used to study the organization of biofilms [75]. The effect of NaCl on *Bacillus* strains has only been studied at higher concentrations, which showed a decrease in both biofilm formations and motility [47,66,76–78]. Our study used the physiologically relevant concentrations normally found in the environment where *B. subtilis* is profusely found, i.e. associated with plants and soil [52,79,80]. By varying the concentration of NaCl, a reduction in wrinkles, the pellicle dry weight and carbohydrate content, and reduced hydrophobicity, cumulatively leading to a major change in the biofilm architecture and properties. While the pellicle-forming ability is considerably decreased upon exposure to NaCl, we observed a rapid expansion of biofilms on agar surfaces, which is due to the increase in the percentage of motile subpopulation of cells within the bacterial biofilms. Concomitantly, at the cellular level, NaCl induced rapid diffusive movement of bacteria along the direction of lateral expansion, in contrast to the slow sub-diffusive behaviour in the absence of NaCl. Thus, our study reveals that NaCl acts as a switch that triggers the sessile to an independent unicellular motile state transition. Through quantitative RT PCR, we observed the upregulation of motility genes and the downregulation of biofilm genes, thereby highlighting the molecular mechanisms underlying the observed behaviour.

Notably, we unravel the upregulation of motility-associated genes alongside a downregulation of key biofilm formation genes, unveiling a dynamic interplay that governs the transition from sessile to motile states. Furthermore, the architectural and gene expression changes induced by NaCl are specifically mediated by the cellular uptake of salt through sodium ion channels. This suggests that NaCl can reprogramme gene expression and alter cellular morphology and the state of cells to adapt to independent unicellular motile cells, which may facilitate bacterial colony escape or expansion.

## Data Availability

All the data are presented in the main manuscript and supplementary material [81].
